# Cryopreservation of primary human monocytes does not negatively affect their functionality or their ability to be labelled with radionuclides: basis for molecular imaging and cell therapy

**DOI:** 10.1186/s13550-016-0232-5

**Published:** 2016-10-24

**Authors:** Evangelia Pardali, Timo Schmitz, Andreas Borgscheiper, Janette Iking, Lars Stegger, Johannes Waltenberger

**Affiliations:** 1 Department of Cardiovascular Medicine, University Hospital Münster, Albert-Schweitzer-Campus 1, Building A1, 48149 Münster, Germany; 2 Department of Nuclear Medicine, University Hospital Münster, 48149 Münster, Germany; 3 Cells-in-Motion Cluster of Excellence (EXC 1003 - CiM), University of Münster, 48149 Münster, Germany

**Keywords:** Monocytes, Cryopreservation, Migration, Adhesion, Radionuclide labelling

## Abstract

**Background:**

Circulating white blood cells crucially contribute to maintenance and repair of solid organs. Therefore, certain cell populations such as monocytes are attractive targets for use in molecular imaging and cell imaging, e.g. after labelling with radionuclides, as well as for cell therapies. However, the preparation of monocytes may require freezing and thawing to preserve cells for timely and standardised applications. Additional modifications of these cells such as radioisotope labelling are necessary prior to their application in vivo. We therefore tested the hypothesis whether cryopreservation of freshly isolated circulating human monocytes affects their functional phenotype or their suitability for radionuclide labelling.

**Results:**

CD14+CD16− monocytes were isolated from human peripheral blood. They were either directly used for cellular assays and labelling or frozen down using cryoprotectants. In the latter case, cells were thawed prior to further use and analysed for survival, chemotactic responses to various growth factors and adhesion on endothelial cells. In addition, both fresh and cryopreserved monocytes were labelled with radiotracers followed by assessment of survival and chemotactic responses. In all functional assays performed, cryopreserved monocytes did not significantly differ from freshly isolated monocytes with regard to their functionality. Cryopreservation did not affect cell survival. There was no effect on the chemotactic response of monocytes towards different growth factors. Likewise, adhesion properties remained unchanged following cryopreservation. Moreover, the labelling efficiency was similar for freshly isolated and cryopreserved monocytes. Labelling did not negatively affect monocyte survival and function.

**Conclusions:**

Our data indicate that cryopreservation of freshly isolated human primary monocytes is feasible and does not negatively affect their functionality when used for labelling and functional assessment.

## Background

Monocytes play an important role in inflammation and in host defence as they participate in both innate and adaptive immune responses. They are circulating mononuclear phagocyte-like cells which are derived from a common myeloid precursor in the bone marrow [1]. Although they do not proliferate outside the bone marrow, monocytes circulate for several days in the blood stream to peripheral tissues where they patrol blood vessels until they enter tissues and differentiate or until they die [2–4]. In addition, undifferentiated monocytes can reside in the subcapsular red pulp of the spleen in a much higher concentration than in the circulation [5]. Monocyte recruitment and infiltration into tissues are modulated by proinflammatory as well as metabolic stimuli. Upon tissue infiltration, monocytes can further differentiate irreversibly into tissue macrophages and/or dendritic cells following stimulation by different factors or induced by inflammation [4, 6].

Peripheral blood monocytes represent 5–10 % of the human leukocytes in the peripheral blood. They show morphological heterogeneity and can be categorised in different subclasses depending on the expression of CD14, which is part of the lipopolysaccharide (LPS) receptor, and CD16, also known as the FcγRII receptor [7, 8]. Human monocytes were classified into classical CD14^++^CD16^−^ (85–90 % of monocytes) and non-classical CD14^+^CD16^++^ monocytes (10–15 % of the total monocytes). As a third subset, the intermediate CD14^++^CD16^+^ monocytes was characterised [9]. Monocytes play crucial roles in the clearance of bacteria, viruses and toxic substances and in the eradication of apoptotic and necrotic cells. In addition, monocytes participate in the regulation of angiogenesis and arteriogenesis as well as tissue repair following injury [10].

However, increased accumulation of monocytes appears in inflammatory diseases such as rheumatoid arthritis, atherosclerosis, diabetes, hypertension, myocardial infarction and other cardiovascular diseases or cancer [7, 11, 12]. Thus, the ability of monocytes to mobilise and migrate to the sites of inflammation, angiogenesis/arteriogenesis and tissue repair is of great importance. Moreover, monocytes are the inflammatory cell type dominating the infarcted myocardium as they play an important role in heart repair as well [10, 13]. Moreover, monocytes may represent a therapeutic target after acute myocardial infarction since increased accumulation of monocytes lead to increased inflammation in the infarcted hearts [10, 13]. So far, no data is available for human monocyte mobility and accumulation following myocardial infarction although different studies have focused on the characterisation of mouse monocyte mobility [13].

Chemotaxis, i.e. directed migration, is a crucial aspect of monocyte function. Several studies have shown that CD14^++^CD16^−^ monocytes from patients with cardiovascular risk factors, such as diabetes mellitus, hypertension, smoking or hypercholesterolemia, show a chemotactic defect towards vascular endothelial growth factor-A (VEGF-A) and monocyte chemoattractant protein-1 (MCP-1) ligands [14–20]. However, it remains unknown how their chemotactic responses are affected in vivo and whether this has an effect on the development of cardiovascular diseases, arteriogenesis and cardiovascular healing.

Monocytes are attractive targets for use in molecular and cellular imaging as well as for cell therapy due to their important role in vascular repair and cardiovascular diseases. Cellular imaging of monocytes will allow their detection in the myocardium and their localization within the infarct zone, the border zone or the remote myocardium. In addition, cellular imaging would answer the question whether the phenotype of circulating monocytes in the blood stream correlates with the phenotype of monocytes in the atherosclerotic lesions or in the infarcted myocardium. Thus, techniques for tracking and localising human monocytes in vivo would be of major importance. Targeted imaging approaches require time-consuming modification of these cells prior to their application in vivo and imaging for a prolonged time (hours or more). The process of harvesting human cells from patients with acute myocardial infarction, analysis of cell function, radiolabelling of monocyte subfractions, administration of cells into mice and finally performing imaging with either planar and tomographic (single-photon emission computed tomography (SPECT)) scintigraphy or positron emission tomography (PET) is a long process. As a consequence, imaging must be performed outside office hours (i.e. in the late evening or during the night) under suboptimal working conditions (e.g. due to lack of imaging personnel). Therefore, intermediate freezing of the cells would be very beneficial for timely and standardised application. We therefore tested the hypothesis whether freezing and thawing, i.e. cryopreservation of circulating human monocytes affects the functional phenotype of these cells or their suitability for radioactive labelling.

## Methods

### Monocyte isolation from peripheral human blood

Monocytes were isolated from fresh human blood leukocyte reduction chamber of platelet apheresis sets enriched with white blood cells and platelets from healthy subjects recruited by the blood bank of the University Hospital Münster. The study was approved by the scientific and ethics committee of the University of Münster and conforms to the principles of the Declaration of Helsinki. Written informed consent was obtained from all subjects. Mononuclear cells (MNCs) were obtained from the blood by density centrifugation using Leucosep® tubes (50 ml, with filter, Greiner) and the density gradient media Histopaque® 1077 (Sigma) separation solution. “MACS Monocyte Isolation Kit II” (Miltenyi) was used for isolation of CD14^++^CD16^−^ monocyte subpopulation. Following manufacturer’s instructions, non-monocytes were magnetically labelled using a cocktail of biotin-conjugated antibodies and anti-biotin MicroBeads. Depletion of these magnetically labelled cells was done through magnetic separation. Isolated monocytes were counted using CASY technology and washed once with serum-free RPMI-1640 medium before used for adhesion assay.

### Cryopreservation and subsequent thawing of human monocytes

Monocytes were frozen for future use by suspending 10 × 10^6^ cells/ml in ice cold freezing medium CTS® Synth-a-Freeze® Medium (Gibco). One-millilitre aliquots were dispensed to Biofreeze vials (Stratagene). The cells were transferred to a cryofreezing container (Mr. Frosty, Nalgene) and placed in a −80 °C freezer. On the following day, the vials were transferred to liquid nitrogen and stored there for a duration of 2 days up to 4 weeks before use. The cells were thawed quickly in a 37 °C water bath and transferred into ten volumes of pre-warmed RPMI medium supplemented with 10 % fetal bovine serum (FBS). Trypan blue exclusion indicated that the cells were >95 % viable just after thawing.

### Monocyte chemotaxis assays

Chemotaxis was analysed as previously described [21]. Briefly, freshly isolated human monocytes or cryopreserved monocytes were resuspended in medium at a concentration of 0.5 × 10^6^ cells/ml. Subsequently, monocytes were placed to the upper wells of a chemotactic chamber (Neuroprobe) and migrated towards chemotactic growth factors which were added to the lower wells of the chamber. Upper and lower wells were separated by a polycarbonate membrane (pore size 5 μm, Millipore). The cells were allowed to migrate for 90 min in a humidified incubator (5 % CO_2_) at 37 °C. Adherent cells on polycarbonate membrane were fixed for 10 min using absolute ethanol and stained with Giemsa dye. The non-migrated cells from the upper side of the membrane were scraped off gently with a cotton bud. Migrated cells were quantified by counting cells in five high-power fields (20× primary magnification) of four different wells per condition.

### Human umbilical vein endothelial cells

Human umbilical vein endothelial cells (HUVECs) were isolated from anonymized donors according to the Declaration of Helsinki and approved by the ethics boards of the University of Münster (2009-537-f-S). HUVECs were cultivated as described before [22] on gelatin (1 % in phosphate buffer saline (PBS)) coated T25 and T75 flasks in M199 (containing 20 % FBS, 0.675 % bovine pituitary extract (BPE), 0.125 % heparin and 1.25 % penicillin/streptomycin).

### Adhesion assays

The ability of human monocytes to bind to HUVECs was measured using previously reported methods [23]. Endothelial cells cultured in 48-well dishes were either left unstimulated or stimulated for 4 h with 10 ng/ml human tumour necrosis factor α (hTNFα) in M199 medium containing 1 % FBS at 37 °C. Human primary monocytes were labelled with 4 pg/ml calcein (Invitrogen) at 37 °C for 15 min. Cells were washed once to remove excess of calcein and resuspended in RPMI medium at a concentration of 2 × 10^5^ cells/ml. Prior to the adhesion assay, HUVECs were rinsed three times, and 1 × 10^5^ labelled monocytes per well were added. After 20 min, unbound monocytes were removed and the cells were washed three times with 1× PBS to remove non-adherent monocytes. Pictures were taken in the middle of each well using a 5× objective, light at 490 nm with a Leica Microsystems DMI 3000B microscope, a shutter Leica Microsystems EL6000 and image software LAS V3.7. The number of cells was analysed by ImageJ 1.45s (NIH) with the particle analysis plug-in.

### Cell viability

Cells were seeded in a 96-well plate at a density of 100,000 cells per well in RPMI medium. Cell density was determined every day using the CellTiter 96® AQueous cell proliferation assay (MTS, Promega) according to the manufacturer’s instructions. Briefly, 20 μl of reagent was added to the test wells and incubated at 37 °C for 1 h in 5 % CO2. Optical density was measured at 490 nm by spectrophotometric analysis and viability was calculated as sample optical density divided by control optical density. As a negative control, 150 μl of RPMI medium and 20 μl MTS were used. In addition, the cells were stained with trypan blue and evaluated using light microscopy. The viability observed was always more than 80 %. All assays were performed in triplicates.

### Labelling of monocytes with ^99m^Tc-HMPAO

^99m^Technetium-hexamethylpropyleneamineoxime (^99m^Tc-HMPAO) was prepared using the stabilised Ceretec cobalt labelling kit (GE Healthcare, Buchler GmbH & Co. KG, Braunschweig, Germany) according to the manufacturer’s instructions. Fresh and cryopreserved monocytes (7 × 10^6^/sample) were resuspended in 100 μl of 0.9 % NaCl. Per sample, 150 MBq of ^99m^Tc-HMPAO was added and cells were incubated at room temperature for 20 min. Cells were spun down at 1100 rpm for 10 min and subsequently washed once with 1× PBS. Radioactivity was measured using an ISOMED dose calibrator (MED Nuklearmedizin-Technik Dresden GmbH, Dresden, Germany). Labelling efficiency (LE) was estimated using the following formula: LE = [C / (C + W)] × 100, where C is activity associated with the cells and W is activity associated with the wash.

### Reagents

Recombinant human vascular endothelial growth factor-A (VEGF-A) and placenta growth factor-1 (PlGF-1) were obtained from RELIA*Tech* GmbH (Braunschweig, Germany). Recombinant human transforming growth factor β1 (TGFβ1) and monocyte chemotactic protein-1 (MCP-1) were obtained from Peprotech GmBH (Hamburg, Germany). RPMI 1640 medium was purchased from Invitrogen (Karlsruhe; Germany) and fetal calf serum (FCS) from Biochrom AG (Berlin, Germany). Cell Titer 96® non-radioactive cell proliferation assay was purchased from Promega (Madison, WI, USA). Histopaque separation solution was obtained from Sigma-Aldrich (Saint Louis, MO, USA).

### Statistical analysis

Results are expressed as mean ± SEM using GraphPad Prism (Version 5). To estimate the level of significance, a one-way ANOVA non-parametric Kruskal-Wallis test for unpaired samples with Dunn’s post test or the Mann-Whitney test were used. A probability (*p*) value of <0.05 was considered statistically significant. All calculations were performed using SPSS version 22 (*p < 0.05, **p < 0.01, ***p < 0.001).

## Results

### Effects of cryopreservation on mononuclear cell viability

**Fig. 1:**
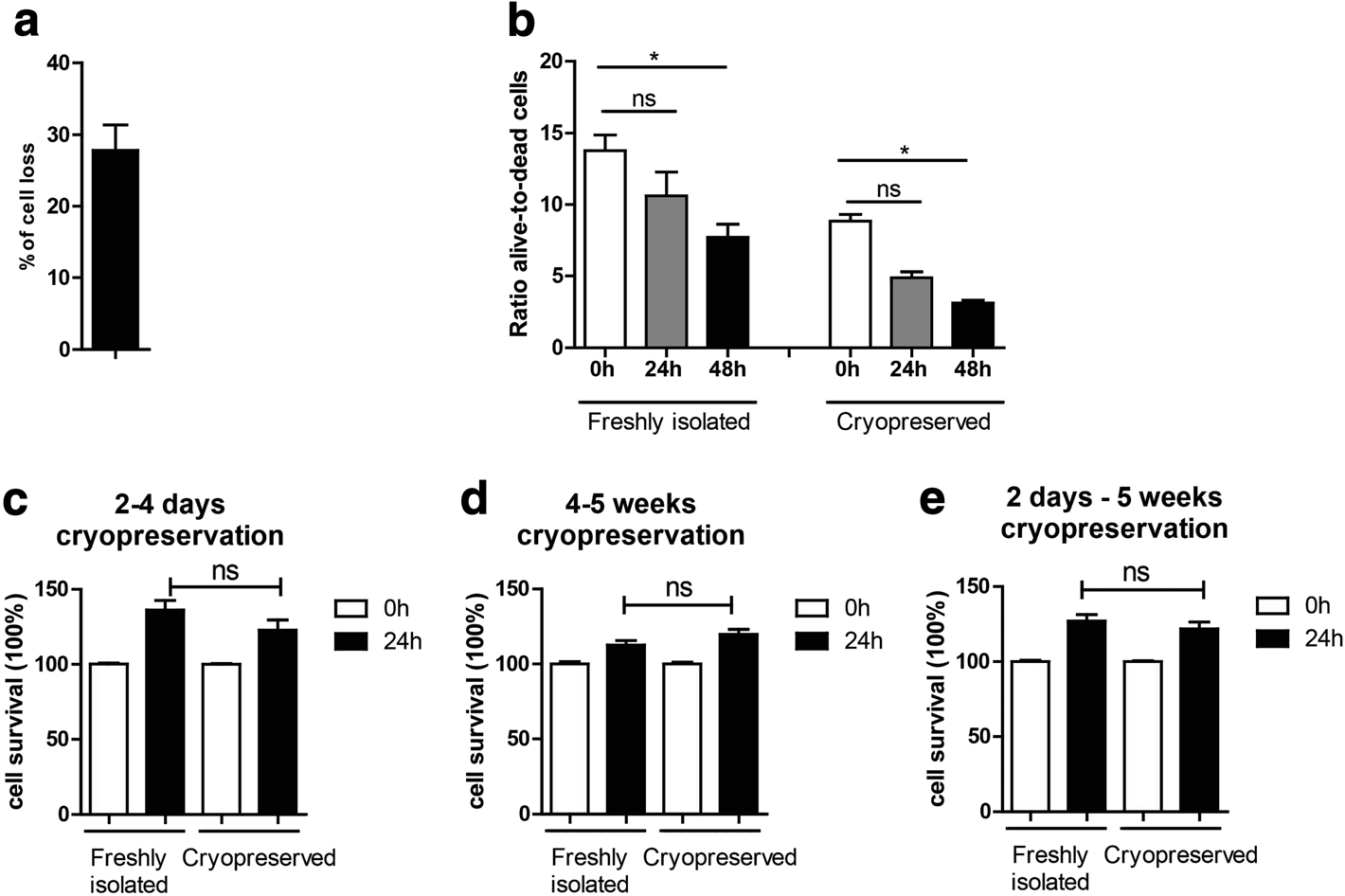
Effects of cryopreservation on viability of CD14^++^CD16^−^ monocytes. **a** Recovery of cryopreserved human monocytes after thawing. **b** Freshly isolated and cryopreserved cells were analysed for their survival using a trypan blue exclusion method. Relative ratios of the alive-to-dead freshly isolated and cryopreserved monocytes were calculated. **c**–**e** Freshly isolated and cryopreserved cells were analysed with the MTS viability assay at 0 h, 24 h after isolation or thawing, 2–4 weeks (*n* = 7) or 4–5 weeks (*n* = 10) after cryopreservation. Statistics: Kruskal-Wallis test with Dunn’s post test, *p* value: *p < 0.05

First, we characterised the effects of cryopreservation on the recovery of cryopreserved CD14^++^CD16^−^ monocytes. As shown in Fig. 1a, cryopreservation resulted in 27.8 ± 12 % loss of monocytes after thawing. The viability of both fresh and cryopreserved cells was tested using a trypan blue exclusion assay. As shown in Fig. 1b, cell cryopreservation had no statistically significant effect on the ratio between alive and dead cells at 24 h; however, the difference reaches statistical significance after 48 h both for freshly isolated and cryopreserved cells. Nevertheless, considering that the cryopreserved cells will be labelled and administrated immediately after thawing for live cell imaging shortly after injection into the recipient, the small reduction in number of living cells at 48 h post thawing may not affect the imaging results.

We further analysed the effects of cryopreservation on survival of human monocytes using the MTS CellTiter 96 AQueous One Solution cell proliferation assay. The data suggest that cryopreservation for short (2–4 days) or longer periods of time (4–5 weeks) did not affect survival of CD14^++^CD16^−^ monocytes (Fig. 1c–e). Nevertheless, there is a slight increase in proliferation/survival of monocytes at 24 h. The MTS compound is processed by cells into a coloured formazan product that is soluble in tissue culture medium. This conversion is presumably accomplished by NADPH or NADH produced by dehydrogenase enzymes in metabolically active cells. Thus, the small increase in the survival at 24 h may reflect increased metabolic activity of the cells at 24 h post isolation (freshly isolated monocytes) and 24 h after thawing (cryopreserved monocytes) and does not relate with monocyte proliferation.

### Chemotactic responses of fresh versus cryopreserved human monocytes

**Fig. 2:**
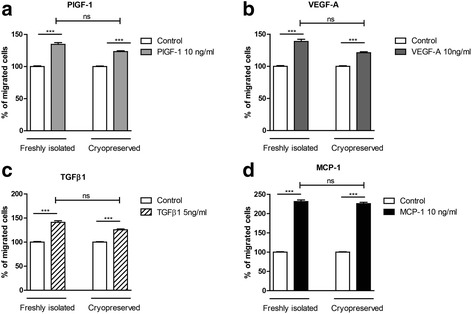
Effects of cryopreservation on chemotaxis of CD14^++^CD16^−^ monocyte. **a**–**d** Freshly isolated and cryopreserved monocytes were analysed for their chemotactic responses towards different concentrations of growth factors including PlGF1, VEGF-A, TGFβ and MCP-1 using a modified Boyden chamber chemotaxis assay (*n* = 20). Statistics: Kruskal-Wallis test with Dunn’s post test, *p* value: ***p < 0.001

One important aspect of monocyte function is chemotaxis, i.e. directed migration to sites of inflammation, neoangiogenesis or collateral growth, i.e. arteriogenesis. The effects of cryopreservation on mononuclear cell migration was analysed by comparing the migratory responses of freshly isolated and cryopreserved monocytes to various concentrations of the growth factors PlGF-1, VEGF-A, TGFβ1 and MCP-1 (Fig. 2). The results (*n* = 20) demonstrate that both fresh and cryopreserved monocytes show significant migratory responses towards the different chemoattractants (Fig. 2a–d). Although there is a small reduction (5–15 %) of the chemotactic responses in cryopreserved monocytes, there is no significant difference (*p* > 0.05) when compared to freshly isolated monocytes (one-way ANOVA Kruskal-Wallis test with Dunn’s post test).

### Cryopreservation does not affect monocyte adherence to endothelial cells

**Fig. 3:**
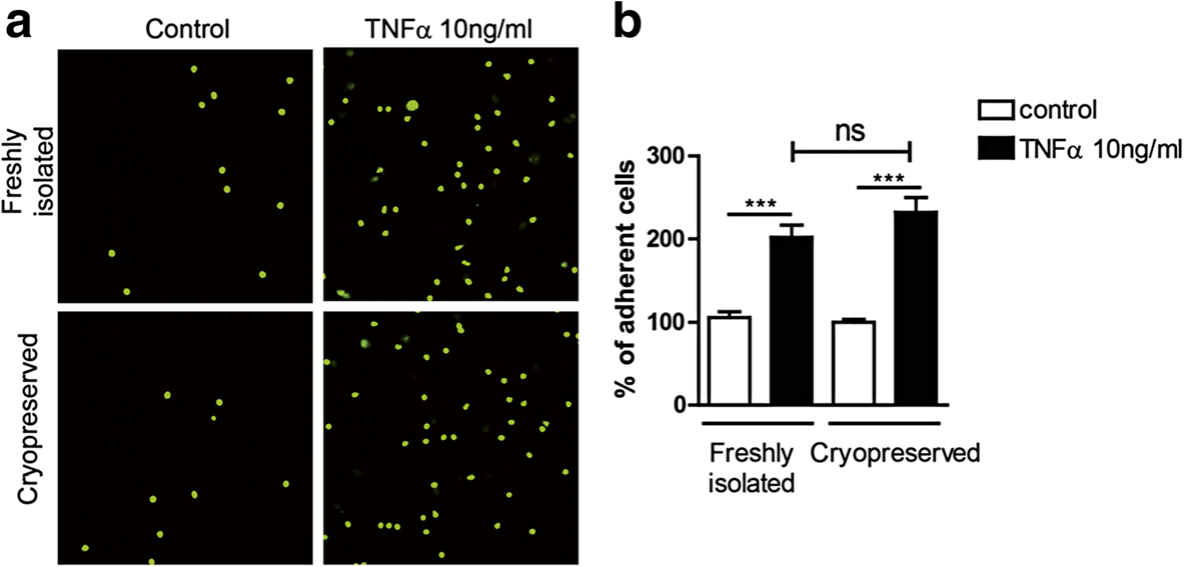
Effect of cryopreservation of CD14^++^CD16^−^ monocytes on adhesion to endothelial cells. **a** Freshly isolated and cryopreserved primary monocytes were allowed to adhere on TNFα stimulated or unstimulated HUVECs. **b** The number of adherent cells was quantified (*n* = 6). Statistics: Kruskal-Wallis test with Dunn’s post test, *p* value: ***p < 0.001

An important aspect of monocyte function is their adherence to the endothelium, which in turn is the initial step of monocyte recruitment to inflammatory sites, followed by transmigration through the endothelium. Therefore, we analysed the effect of cryopreservation on monocyte adhesion to HUVECs. Adhesion of cells on unstimulated endothelial cells was set as 100 %. Our results (*n* = 6) suggest that both freshly isolated (100 and 202 %) as well as cryopreserved monocytes (100 and 232.2 %) adhere to both non-activated and TNFα-activated HUVECs in a similar fashion (Fig. 3) and that there are no significant differences (*p* > 0.05) in their adhesion.

### Cryopreservation of human monocytes does not affect their labelling with the radiopharmaceutical ^99m^Tc-HMPAO or their cellular function

Since monocytes are putative targets for use in molecular and cellular imaging, there is an urgent need for the development of novel approaches (e.g. the establishment of specific protocols) to study mononuclear cell trafficking in vivo. We sought to study whether labelling of human monocytes with radiopharmaceuticals for cell tracking in vivo is efficient and whether labelling does affect their function. Experiments were performed both for fresh unfrozen cells and for cryopreserved cells. We have chosen the radiopharmaceutical ^99m^Tc-HMPAO which is established for cell labelling and suitable for planar and tomographic scintigraphy.

**Fig. 4:**
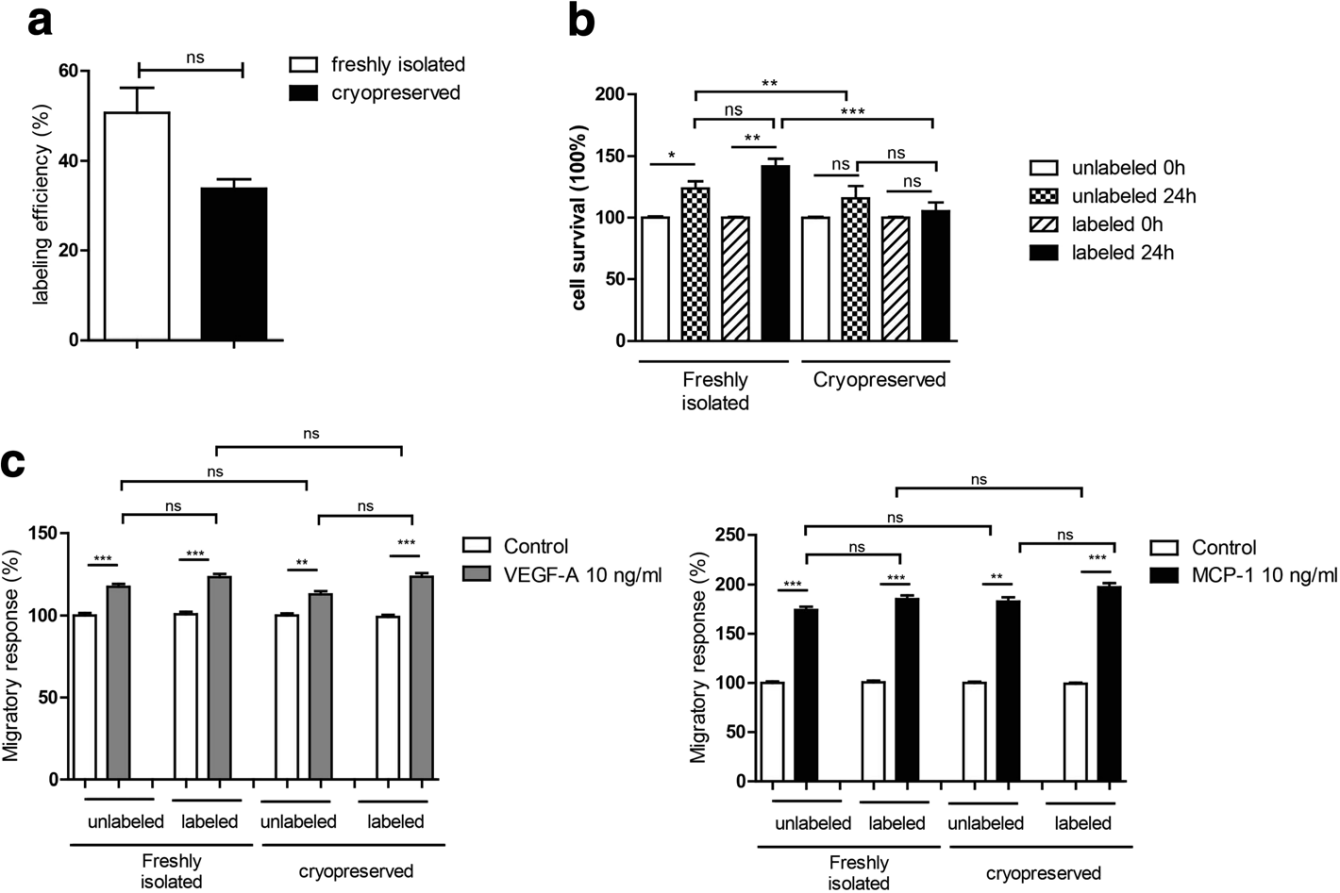
Effects of cryopreservation on monocyte ^99m^Tc-HMPAO labelling and functionality. Freshly isolated and cryopreserved monocytes were radiolabelled with 150 MBq ^99m^Tc-HMPAO and analysed **a** for the efficiency of labelling, **b** for their survival using the MTS viability at 0 and 24 h after labelling and **c** for their migratory responses towards VEGF-A (10 ng/ml) and MCP-1 (10 ng/ml) in a modified Boyden chamber chemotaxis assay (*n* = 12). Statistics: Kruskal-Wallis test with Dunn’s post test, or the Mann-Whitney test was performed, *p* value: *p < 0.05; **p < 0.01; ***p < 0.001

Labelling of fresh CD14^++^CD16^−^ monocytes with ^99m^Tc-HMPAO resulted in labelling efficiency (LE) of 50.6 % (Fig. 4a). Labelling did not affect cell survival of mononuclear cells as measured by the MTS cell survival assay (*n* = 12, *p* > 0.05; Fig. 4b). Moreover, we analysed the effects of ^99m^Tc-HMPAO labelling on chemotactic responses. Basal migration of cells was set as 100 %. Migration towards VEGF-A and MCP-1 was consistent for both labelled (117.5 and 174.27 %) and non-labelled monocytes (123.4 and 185.4 %). No significant difference to unlabelled cells could be detected (*n* = 12, *p* > 0.05; Fig. 4b). These results suggest that ^99m^Tc-HMPAO labelling of primary human monocytes does not strongly interfere with mononuclear cell viability and function.

Next, we studied the effects of cryopreservation on monocyte labelling. Cryopreserved human CD14^++^CD16^−^ monocytes were labelled with ^99m^Tc-HMPAO. Labelling of cryopreserved monocytes with HMPAO resulted in a LE of 33.8 % (Fig. 4a). The difference of LE of fresh and cryopreserved cells did not reach statistical significance (*n* = 12, *p* > 0.05). ^99m^Tc-HMPAO labelling did not affect viability of cryopreserved mononuclear cells as measured by the MTS cell survival assay (Fig. 4b). Cryopreserved labelled monocytes migrated efficiently towards VEGF-A and MCP-1 and showed no significant differences to unlabelled cells (Fig. 4c). These results suggest that ^99m^Tc-HMPAO labelling of cryopreserved monocytes is as efficient as using freshly isolated monocytes as labelling does not interfere with mononuclear cell viability and function.

## Discussion

Monocytes are attractive targets for use in cellular imaging and for cell therapies as they play important roles in vascular repair and cardiovascular diseases. Both concepts require the ex vivo modification of cells prior to their application in vivo. An intermediate freezing step may be very beneficial to preserve cells for timely and standardised application in order to optimise imaging experiments, e.g. by avoiding imaging during off hours. For this, we sought to investigate whether freezing and thawing of circulating primary human monocytes affects the functional phenotype of these cells and their suitability for radioactive labelling.

Our results suggest that cryopreservation of primary human CD14^++^CD16^−^ monocytes does not interfere with their survival as cryopreserved monocytes showed no deficits in viability compared to freshly isolated monocytes. In addition, our data show that the chemotactic responses of cryopreserved monocytes towards different concentrations of various growth factors are not negatively affected. Our results suggest that cryopreserved monocytes retain their ability to adhere to quiescent as well as activated endothelium to similar levels as freshly isolated monocytes do. Thus, primary monocytes can be isolated safely at any time point, cryopreserved and stored for later use. Moreover, cryopreserved monocytes can be used for functional testing such as endothelial adhesion assays and migration assays along with isolates from other types of patients and controls. Our results are in line with previous studies which demonstrated that cryopreservation of human monocytes does not affect viability, migration or differentiation of monocytes into macrophages and dendritic cells, IL6 secretion and cryopreserved monocyte could be used efficiently in the monocyte activation test (MAT) [24, 25].

Our study has shown that cryopreservation of human CD14^++^CD16^−^ monocytes does not interfere with their suitability for radiolabelling with ^99m^Tc-HMPAO for subsequent cell imaging. In fresh and cryopreserved monocytes, labelling with 150 MBq ^99m^Tc-HMPAO does not affect mononuclear cell survival or their chemotactic responses The labelling efficiency was lower in our study for cryopreserved cells when compared to fresh cells, albeit not significantly. Cellular accumulation and retention of ^99m^Tc-HMPAO relies on (1) the conversion of the lipophilic ^99m^Tc-HMPAO complex into a hydrophilic complex. and 2) on the redox state of cells or the reaction of ^99m^Tc-HMPAO with sulphydryl reagents like glutathione inside the cell [26–28]. It has been suggested that intracellular conversion of the hydrophobic Tc-HMPAO to a species which is incapable of rapid back diffusion involves interaction of ^99m^Tc-HMPAO with glutathione. [29]. It was shown that cryopreservation of human and rat hepatocytes and human sperm results in reduced glutathione content and glutathione-conjugating capacity [30, 31]. Based on these studies we could suggest that the slightly reduced labelling efficiency of cryopreserved human monocytes is due to reduced glutathione levels.

The establishment of a protocol to cryopreserve and revitalise primary human monocytes, which can be radiolabelled while retaining their viability and their functionality, represents a major advancement for both cellular imaging and cell therapy. This will allow the systematic usage of these cells as well as the comparison of cells from different patients. It has previously been shown that monocytes from either diabetes mellitus or hypercholesterolemic patients exhibit defective chemotactic responses to individual growth factors ex vivo including VEGF-A or MCP-1 [14–17, 20, 32]. It remains unclear whether reduced growth factor induced chemotactic responses are relevant for mononuclear cell function in vivo and to the development of atherosclerosis and/or to effective vascular healing in patients.

Based on the ability of monocytes and macrophages to phagocytose, several approaches have been developed for their targeted imaging in vivo. For example, different types of nanoparticles labelled with isotopes such as fluorine-18 [33] and copper-64 [34] or ultra-small superparamagnetic iron oxide (USPIO) nanoparticles were used to image monocytes/macrophages with positron emission tomography (PET) and magnetic resonance imaging (MRI), respectively [35]. In addition, the standard PET tracer 2-deoxy-2-[18F]fluoro-D-glucose ([^18^F]FDG), a glucose analogue, is taken up in vivo after intravenous injection by cells with a high metabolic rate such as mononuclear cells. It has been used for imaging inflammation of atherosclerotic plaques in coronary arteries [36–38]. Nevertheless, [^18^F]FDG is not specific for leukocytes in the atherosclerotic plaque. The same tracer could be used for in vitro labelling of inflammatory cells before injection for tracking mononuclear cells in vivo. However, [^18^F]FDG leakage out of these labelled cells would interfere with imaging specificity. It is thus of great importance to develop methods which will allow us to track and study primary human monocytes from different patients in vivo. The establishment of a method to cryopreserve and later efficiently label human monocytes ex vivo will allow us to study their localisation in vivo as well as their function and contribution to vascular healing in preclinical mouse models of angiogenesis and arteriogenesis. This way, we will gain more insights into the mononuclear cell dysfunction and how this translates into their role in vivo as well as their contribution to the development of vascular diseases.

Monocytes play an important role in myocardial infarction healing. However, they may have damaging effects on the myocardium in the early and late reperfusion period as well [10, 13, 39]. Therefore, imaging approaches to detect increased and sustained inflammation post MI will be of great importance for estimating the effect of monocytes on myocardial damage and repair. Moreover, ex vivo labelled monocytes can be used to localise them in vivo within the infarcted myocardium and the border zone. This may reveal hints to appreciate their potential contribution to post-infarct healing in preclinical mouse models as well as in consecutive clinical studies for in vivo trafficking of mononuclear cells in the human post-infarcted heart.

## Conclusions

Taken together, our work demonstrates that freshly isolated and then cryopreserved monocytes, subsequently thawed and labelled with the radiotracer ^99m^Tc-HMPAO, retain all functional properties that have been tested so far. Therefore, monocyte cryopreservation as documented in our approach is feasible and will benefit studies on monocytes for cellular imaging and cell therapy.

## Abbreviations

[^18^F]FDG: 2-deoxy-2-[18F]fluoro-D-glucose
^99m^Tc-HMPAO: 99mTechnetium-hexamethylpropyleneamineoxime
hTNFα: Human tumour necrosis factor α
HUVECs: Human umbilical vein endothelial cells
LE: Labelling efficiency
MCP-1: Monocyte chemoattractant protein-1
MRI: Magnetic resonance imaging
PET: Positron emission tomography
PlGF-1: Placenta growth factor-1
SPECT: Single-photon emission computed tomography
TGFβ1: Transforming growth factor β1
USPIO: Ultra-small superparamagnetic iron oxide
VEGF-A: Vascular endothelial growth factor-A
